# Safety and efficacy of autologous bone marrow clot as a multifunctional bioscaffold for instrumental posterior lumbar fusion: a 1-year follow-up pilot study

**DOI:** 10.3389/fendo.2023.1245344

**Published:** 2024-01-08

**Authors:** Francesca Salamanna, Giuseppe Tedesco, Maria Sartori, Cristiana Griffoni, Paolo Spinnato, Paolo Romeo, Riccardo Ghermandi, Milena Fini, Gianluca Giavaresi, Alessandro Gasbarrini, Giovanni Barbanti Brodano

**Affiliations:** ^1^ Surgical Sciences and Technologies, IRCCS Istituto Ortopedico Rizzoli, Bologna, Italy; ^2^ Spine Surgery Unit, IRCCS Istituto Ortopedico Rizzoli, Bologna, Italy; ^3^ Diagnostic and Interventional Radiology, IRCCS Istituto Ortopedico Rizzoli, Bologna, Italy; ^4^ Scientific Direction, IRCCS Istituto Ortopedico Rizzoli, Bologna, Italy

**Keywords:** bone marrow clot, multifunctional bio-scaffold, lumbar fusion, degenerative spine disease, pilot study

## Abstract

**Background:**

Bone marrow aspirate (BMA), when combined with graft substitutes, has long been introduced as a promising alternative to iliac crest bone graft in spinal fusion. However, the use of BMA is limited by the absence of a standardized procedure, a structural texture, and the potential for diffusion away from the implant site. Recently, the potential use of a new formulation of BMA, named BMA clot, has been preclinically described. In this report, we present the results of a prospective pilot clinical study aimed at evaluating the safety and efficacy of autologous vertebral BMA (vBMA) clot as a three-dimensional and multifunctional bioscaffold in instrumented posterior lumbar fusion.

**Methods:**

Ten consecutive patients with an indication of multilevel (≤5) posterior spinal fusion due to lumbar spine degenerative diseases were included in the study and treated with vBMA. Clinical outcomes were assessed using the Visual Analog Scale (VAS), Oswestry Disability Index (ODI), and EuroQoL-5L (EQ-5L) preoperatively and at 3 months and 12 months after spinal fusion. Bone fusion quality was evaluated at the 12-month follow-up using the Brantigan classification on radiography (XR) imaging. Bone density was measured on computed tomography (CT) scans at 6 and 12 months of follow-up visits at the intervertebral arches and intervertebral joint areas and expressed in Hounsfield unit (HU).

**Results:**

The results indicate a successful posterolateral fusion rate of approximately 100% (considering levels with C, D, and E grades according to the Brantigan classification) at the 12-month follow-up, along with an increase in bone density from 6 to 12 months of follow-up. An improvement in the quality of life and health status following surgery, as assessed by clinical scores (ODI, VAS, and EQ-5L), was also observed as early as 3 months postsurgery. No adverse events related to the vBMA clot were reported.

**Conclusion:**

This prospective pilot study demonstrates the effectiveness and safety profile of vBMA clot as an advanced bioscaffold capable of achieving posterior lumbar fusion in the treatment of degenerative spine diseases. This lays the groundwork for a larger randomized clinical study.

## 1 Introduction

The use of spinal fusion procedures has rapidly increased over the last decade to help stabilize spines suffering from degenerative, oncologic, and traumatic spine diseases (1), and epidemiological data predict its significant increase in the coming decades (2). Despite advances in spinal surgery, pseudarthrosis still occurs in approximately 25%–35% of procedures (3). These techniques employ autograft or allograft bone tissue, occasionally supplemented with bone substitutes (4). Iliac crest autograft displays fusion rates of over 90%, representing the “gold standard” treatment for spine fusion surgery (5). However, this procedure carries potential complications, such as longer surgical time, donor site morbidity, and acute or chronic pain (6). Local bone graft, retrieved from spinous processes and/or laminae, is an excellent alternative, with fusion rates comparable to those of iliac crest grafts; however, its main drawback is the limited availability (6). In contrast, allografts, which can be used in diverse forms such as freeze-dried, cancellous chips, fresh-frozen, and demineralized bone matrix, are often associated with autografts as they possess only osteoconductive and mild osteoinductive characteristics (7). Contradictory data are presented on their effectiveness when employed alone, with some authors identifying low-fusion rates while others demonstrating analogous outcomes to autografts and fewer adverse events (7, 8). Further evaluations of alternative grafts and materials, such as calcium phosphate ceramics presented in different forms, have taken place; however, the literature indicates that fusion rates with ceramics alone were inadequate because emphasis was placed only on osteoconductive characteristics (9). In this context, the potential therapeutic use of cell therapy approaches for bone repair, specifically adult mesenchymal stromal cells (MSCs), has long been studied (10–14). Sources such as bone marrow (BM), adipose tissue, umbilical cord, and dental-related tissues have been utilized for bone regeneration purposes, and the use of BM-MSCs, the first discovered type, remains to be the most popular today (14). These cells can either be used after being cultured or, simpler, as a one-step procedure, injected as an unprocessed bone marrow aspirate (BMA) or BM concentrate (BMC) (14). The use of cultured BM-MSCs is limited due to the regulation on *in-vitro* cell processing. In contrast, the use of whole BMA or BMC comprises marginal cell manipulation and is further used in clinical practice to treat various musculoskeletal diseases in a “one-step” procedure (15). Although only 0.01%–0.001% of MSCs are present among total mononuclear cells in the BMA, the cohabitation of non-adherent osteogenic cells and the establishment of cell–cell interactions suggested that the use of whole BMAs, instead of BMCs or MSCs expanded and purified, represents a better approach for bone cell therapy (15, 16). During spinal fusion surgery, the vertebral BMA (vBMA) accessed in a routine course of pedicle screw instrumentation resulted to be the best source for marrow aspiration. However, its use is restricted by the absence of an effective processing method and a structural texture and by the possibility of dispersion away from the implant site. In 2018, our group described for the first time the use of a new formulation of vBMA, the vBMA clot (17); MSCs from human clotted vBMA exhibited higher kinetics of growth in comparison to MSCs from human unclotted vBMA as well as higher growth factor expression, higher osteogenic and chondrogenic differentiation ability, and lower expression of TALE and HOX genes, classes of genes that harmfully control osteoblast proliferation, differentiation, and maturation (18). This result was replicated among elderly and super-elderly patients, in whom it was revealed that donor age does not disturb tissue-specific vBMA clot regenerative properties nor the expression of the KLOTHO gene (aging suppressor gene) or senescence-associated genes (18). Collectively, these studies (15–18) suggested that the vBMA clot may play an important role as an osteogenic and osteoinductive 3D bioscaffold, being an abundant source of mesenchymal and hematopoietic stem cells working in a synergic manner to promote bone regeneration. The clotted vBMA use not only removes the need to concentrate and/or purify vBMA but also gives a smart spinal fusion cell therapy approach able to offer high “stability” to the graft site. To substantiate this finding, a prospective pilot clinical study involving 10 patients with degenerative spine disease utilizing clotted vBMA was conducted.

## 2 Methods

### 2.1 Clinical study design

A prospective pilot clinical study was carried out at our center from July 2020 to July 2022, following the approval of the Local Ethics Committee (CE AVEC 587/2020/Sper/IOR). The study was registered with ClinicalTrials.gov (TNR NCT05936047) and carried out in accordance with the principles of the Declaration of Helsinki.

The sample size was calculated considering the standard clinical activities the center typically performed and keeping in mind the consideration in the ensuing paragraph; as such, the number of subjects involved was deemed adequate for assessing the variables under consideration. As this is a non-comparative study, comparisons were only made with literature data under the *Discussion* section.

Consecutive patients with indications of multilevel (≤5) posterior spinal fusion due to lumbar spine degenerative diseases were screened for inclusion in the study, after providing written informed consent. The period of enrolment was from July 2020 to July 2022, and patients were followed up for 12 months.

Specific inclusion criteria included any patients over the age of 18 at the time of surgery with symptomatic degenerative spine disease needing posterior fusion at the lumbar tract, as well as any patients participating in the study who provided informed signed consent. Patient exclusion criteria were applied if they were dealing with any form of local or systemic infections, inflammatory or autoimmune disease, coagulation disorders, tumor diseases, alcohol or drug abuse, and pregnancy or were receiving any type of chemotherapeutic drugs that might interfere with bone regeneration processes and revision surgery.

### 2.2 Surgical procedure

**Figure 1:**
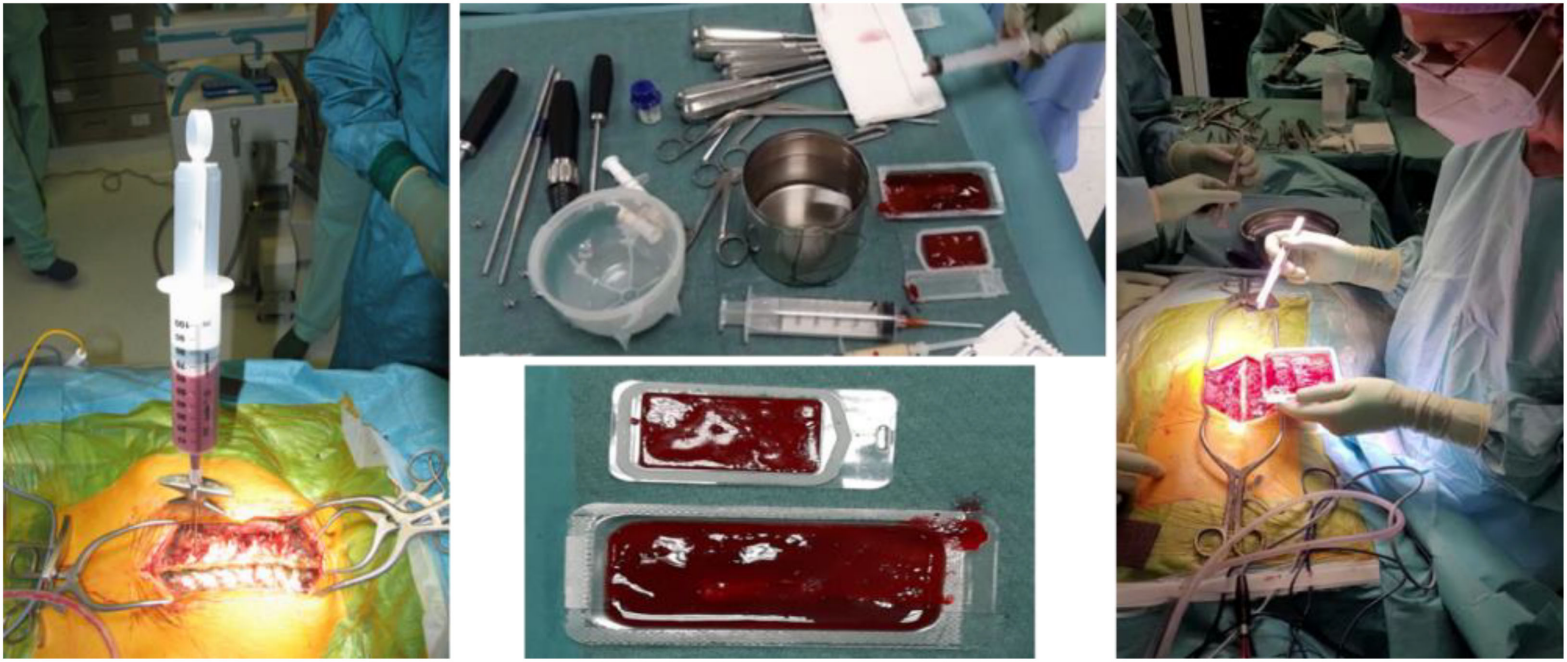
Method used to clot and use the vBMA in the clinical scenario.

A posterior lumbar fusion technique with transpedicular titanium screw/rod instrumentation was performed on all patients. vBMA was harvested from each patient’s vertebral pedicle simultaneously with the preparation of the site for pedicle screw insertion during surgery. In detail, all patients received general anesthesia and were placed in the prone position. Spinal surgery started with a midline skin incision that could be expanded for the adjacent extra levels. Subsequently, subcutaneous dissection beyond the skin incision was carried out in the cranial and caudal directions. Subperiosteal muscle dissection was performed followed by exposure of entry points for pedicle screw and vBMA was harvested. Since the aspiration volume affects the concentration of progenitor cells in vBMA, to maximize their number, a standardized small fraction of marrow of approximately 10–20 ml from each vertebral body (5–10 ml for each pedicle) was aspirated. After aspiration, vBMA was inserted in sterile containers without an anticoagulant and under sterile conditions and allowed to coagulate (clotting time 20–30 min) (**Figure 1**). Meanwhile, the surgical procedure continued. Once the pedicle screws were placed, decompression of the cauda and nerve roots was done with hemilaminectomy and foraminotomy. Subsequently, vBMA was put on the hemilaminae and transverse process on the contralateral side of the hemilaminectomy, following decortication of the lamina, in order to achieve posterolateral fusion. On the hemilaminectomy side, foramino-arthrectomy was done to insert the interbody fusion cage if necessary. vBMA was applied on one side of the vertebra according to the number of segments to be fused.

### 2.3 Radiographic and clinical data analyses

Collection of radiographical and clinical data was carried out prior to surgery and during the 3- and 12-month follow-ups. XR images were employed to determine the fusion degree and bone regeneration by an independent radiologist. The degree of fusion and bone regeneration was assessed at the intervertebral arches and intervertebral joint areas. It was recognized as consisting of a continuous trabecular bone bridge together without radiolucency and scored through the Brantigan classification, which ranks spinal fusion from grade A (pseudoarthrosis) to grade E (certain fusion) (**Table 1**) (19).

**Table 1:** Brantigan classification of spinal fusion.

Classification	Description
A: Obvious radiographic pseudoarthrosis	Pseudoarthrosis, collapse of construct, loss of disc height, vertebral slip, broken screw, displacement of the cage, resorption of bone graft
B: Probable pseudoarthrosis	Significant resorption of bone graft, major lucency or gap visible in the fusion area >2 mm
C: Radiographic status uncertain	A small lucency or gap may be visible with at least half of the graft area showing no lucency between the graft bone and the vertebral bone
D: Probable radiographic fusion	Bone bridges the entire fusion area with at least the density originally achieved at surgery. There should be no lucency between the graft bone and the vertebral bone.
E: Radiographic fusion	The bone in the fusion area is more dense and more mature than originally achieved at surgery; there is no interface between the donor bone and the vertebral bone: a sclerotic line between the graft bone and the vertebral bone indicates a solid fusion. Other indicators of solid fusion are fusion at the facet joints and anterior progression of the graft in the disc.

Even if the Brantigan classification has been introduced to assess intervertebral fusion, it is used also to evaluate posterolateral fusion, which is the goal of our study (20).

Furthermore, bone density was measured on CT scans obtained at 6 and 12 months of follow-up visits, by measuring the fused area of the intervertebral joints with ROI, and expressed in Hounsfield unit (HU). As previously reported (21), we considered grades C, D, and E as successful fusion.

To evaluate the improvement of the patient’s health quality following surgery, clinical scores such as the Oswestry Disability Index (ODI), Visual Analog Scale (VAS), and EuroQoL-5L (EQ-5L) were used (22–24). Standard laboratory parameters of blood samples [e.g., red blood cell count, white blood cell count, platelet count, mean platelet volume, and levels of hemoglobin (Hb)] that were performed preoperatively and again after surgery as well as any comorbidity and adverse event were also systematically collected.

### 2.4 Statistical analysis

The sample size was calculated based on the center’s typical clinical activities and the study’s design (pilot study). Given the small number of patients in the study, no specific statistical analyses were conducted. Consequently, descriptive statistical analysis was used to analyze the clinical scores and CT analysis (VAS, ODI, and EQ-5L).

Results are presented as the number (*n*), mean ± standard deviation, and percentage, as appropriate. After having verified the normal distribution and homogeneity of variance, a one-way ANOVA and multicomparison Tukey’s test were done to identify changes from the baseline to follow-up scores. The level of statistical significance was at *p <*0.05. GraphPad Prism software was utilized (GraphPad Prism 9.5.1).

## 3 Results

### 3.1 Baseline clinical characteristics and demographic data

As reported in **Table 2**, there were seven women (70%) and three men (30%), with a mean age of 49 years (range: 32–65). Most patients were treated for degenerative disc disease (DDD) and one for kyphoscoliosis. Five out of 10 patients received an interbody cage implanted on the side of the hemilaminectomy. Concerning fusion levels, four patients had three, three patients had four, and three patients had five fusion levels. Comorbidity, prevalently hypertension followed by type 1 diabetes, osteoporosis, and hypercholesterolemia, was present in six patients. Most hematological parameters at surgery displayed alterations, with low red blood cell number, Hb, hematocrit, creatinine, and red blood cell distribution width. A high monocyte number was also noted among 9 out of 10 patients. The mean length of stay was 6.2 days (range: 3–12). There was one major complication: a patient with a previous diagnosis of hypertension experienced brady-systolic arrest. According to the surgeon’s surgical notes, this event was not categorized as associated or possibly associated with the BMA clot treatment. No serious adverse events (i.e., immunological reactions, early/late infections, deep wound infection) were recorded.

**Table 2:** Baseline characteristics and demographic data.

Characteristics	Total
***n***	10
***Age at surgery (years), mean ± SD***	49 ± 11.43
***Gender *** * Female* * Male*	** ** 7 3
***Type of diseases *** * Degenerative disc disease* * Kyphoscoliosis*	** ** 9 1
***Type of surgery***	Instrumented posterior fusion
***Levels treated *** * 3* * 4* * 5*	** ** *4* *3* *3*
***Previous surgery***	6
***Smoking***	2
***Comorbidity *** * Hypertension* * Type 1 diabetes* * Osteoporosis* * Hypercholesterolemia*	** ** 3 1 1 1
***Length of stay (day), mean ± SD***	6.2 ± 2.34
***Relevant Lab. hematological parameters at surgery *** * Low red blood cells number* * Low Hb* * Low hematocrit* * High monocytes number* * Low creatinine* * Low red blood cells distribution width*	** ** 5 5 4 3 3 2
***Adverse events *** * Brady-systolic arrest*	* * 1

### 3.2 Follow-up evaluation

All patients were available for clinical and imaging assessment. The degree of spinal fusion assessed by an independent radiologist on radiography performed at 12-month follow-up in all treated levels was evaluated through the Brantigan classification, with a success rate of approximately 100% (considering the levels with C, D, and E grades) (**Table 3**). The posterolateral fusion was assessed at the intervertebral arches and intervertebral joint areas. Taking into account the limited number of patients, no significant differences were observed in the degree of bone fusion between levels with cages and levels without cages.

**Table 3:** Evaluation of bone fusion at the levels treated with BMA clot.

Patient code	Diagnosis	Type of surgery	BMA clot position	Level	Brantigan score right	Brantigan score left
01	Degenerative disc disease	Posterior fusion L3–S1, TLIF L5–S1	Right	L3–L4 L4–L5 L5–S1	D D E	
02	Degenerative disc disease	Posterior fusion L4–S1	Right	L4–L5 L5–S1	D C	
03	Degenerative disc disease	Posterior fusion L4–S1, TLIF L4–L5 and L5–S1	Right	L4–L5 L5–S1	C C	
04	Degenerative disc disease	Posterior fusion T10–S1, TLIF L5–S1	Right	L3–L4 L4–L5 L5–S1	C D D	
05	Degenerative disc disease	Posterior fusion L3–S1, TLIF L4–L5 and L5–S1	Left	L3–L4 L4–L5		C D
06	Degenerative disc disease	Posterior fusion L4–S1, TLIF L4–L5 and L5–S1	Right	L4–L5 L5–S1	E C	
07	Degenerative disc disease	Posterior L2–S1, TLIF L4–L5 and L5–S1	Right	L3–L4 L4–L5 L5–S1	D C C	
08	Adult scoliosis	Posterior fusion T12–L3, Smith Petersen L1–L2 and L2–L3	Right	L1–L2 L2–L3	D D	
09	Degenerative disc disease	Posterior fusion L2–S1	Right	L3–L4 L4–L5 L5–S1	C C D	
10	Degenerative disc disease	Posterior fusion L2–S1, TLIF L4–L5 and L5–S1	Right	L3–L4 L4–L5 L5–S1	E D D	

**Figure 2:**
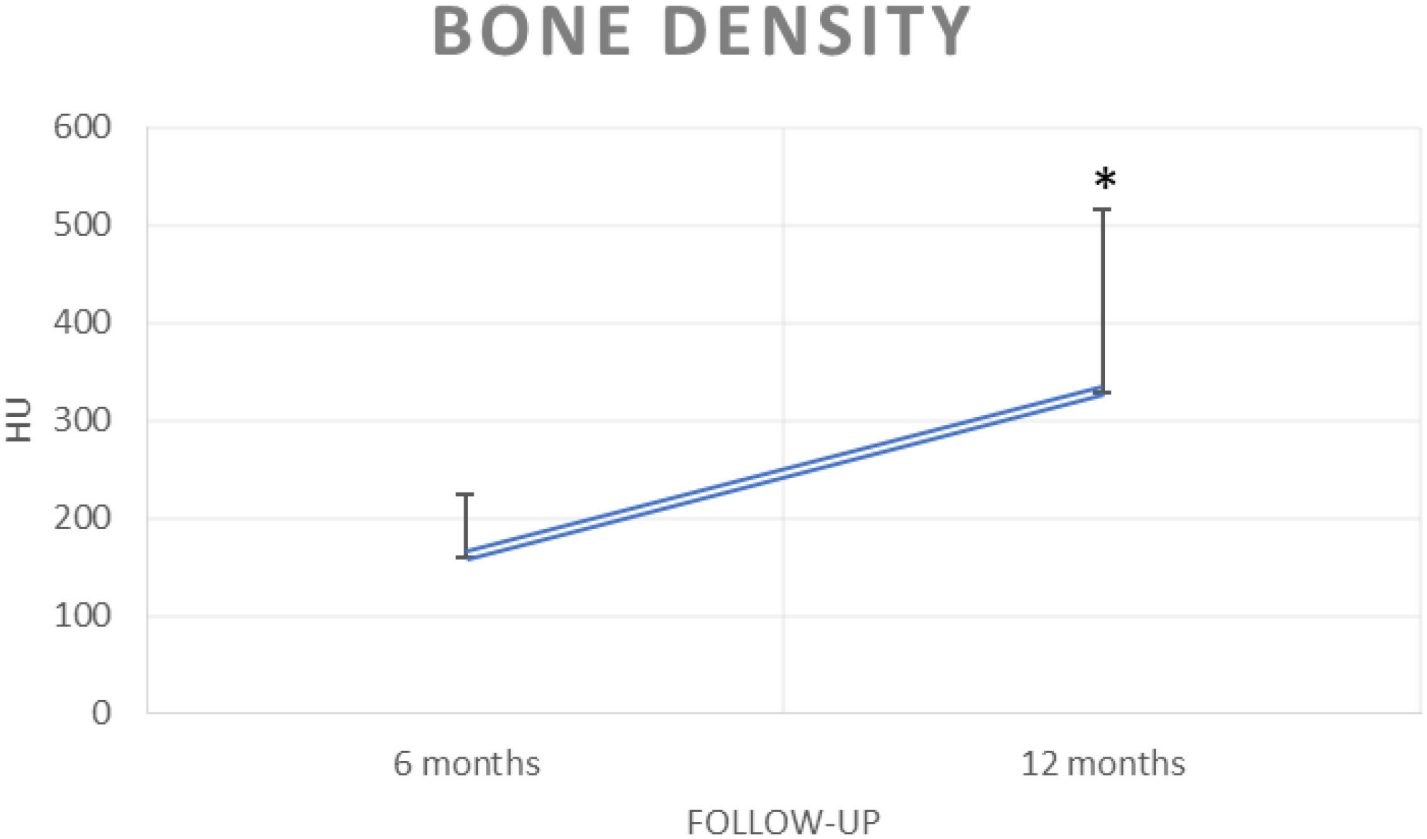
Mean bone density during the follow-up time (*p < 0.05).

The mean bone density evaluated on CT scans increased statistically from 161.4 ± 64.2 HU to 330.6 ± 186.3 HU during follow-up time (*p <* 0.05), as reported in **Figure 2**. The values of bone density reported refer to the levels treated with BMA clot.

In all cases where the posterolateral fusion was performed bilaterally, no significant differences were observed between the degree of bone fusion on the right and left sides, even if on one side the BMA clot was apposed and on the other side the autologous bone graft was used.

**Figure 3:**
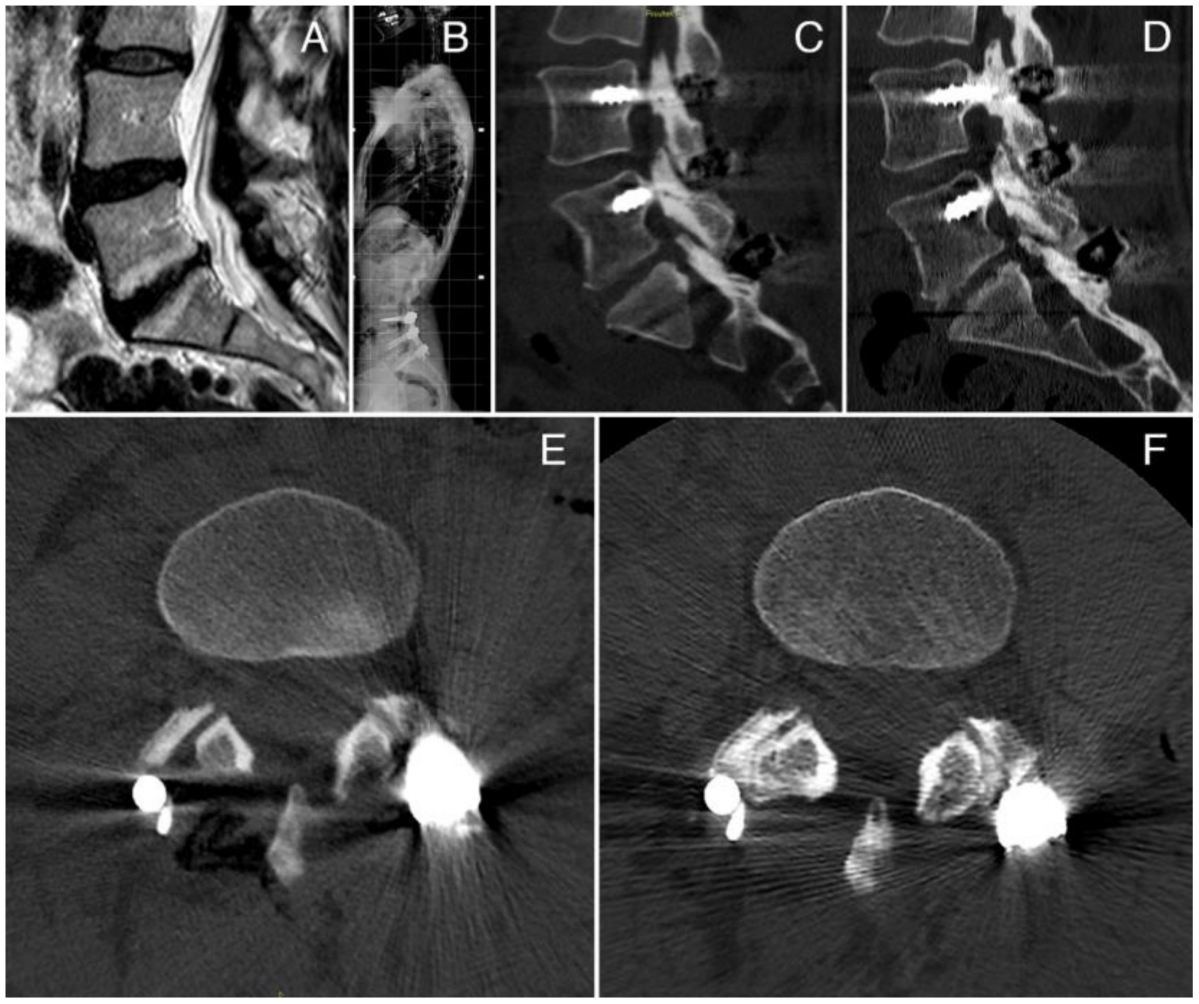
**(A)** Preoperative MRI; **(B)** postoperative X-ray; **(C)** CT scan sagittal view at 6 months of follow-up; **(D)** CT scan sagittal view at 12 months of follow-up, showing a large amount of bone formation at the L4–L5 and L5–S1 levels; **(E)** CT scan axial view at 6 months of follow up; **(F)** CT scan axial view at 12 months of follow-up, showing bone formation at the L4–L5 facet joint level.

One case of spinal fusion with the vBMA clot is reported in **Figure 3**. The case was a 43-year-old woman with a degenerative disc disease (DDD) surgically treated with posterior fusion L4–S1 and vBMA clot. vBMA clot was put on the right side at the L4–L5 level and L5–S1 level. At the 12-month follow-up, a good fusion (grade D) has been detected at the L4–L5 level and at the L5–S1 level (grade C) (**Figures 3D, F**), associated with significant improvement of the clinical outcomes.

**Figure 4:**
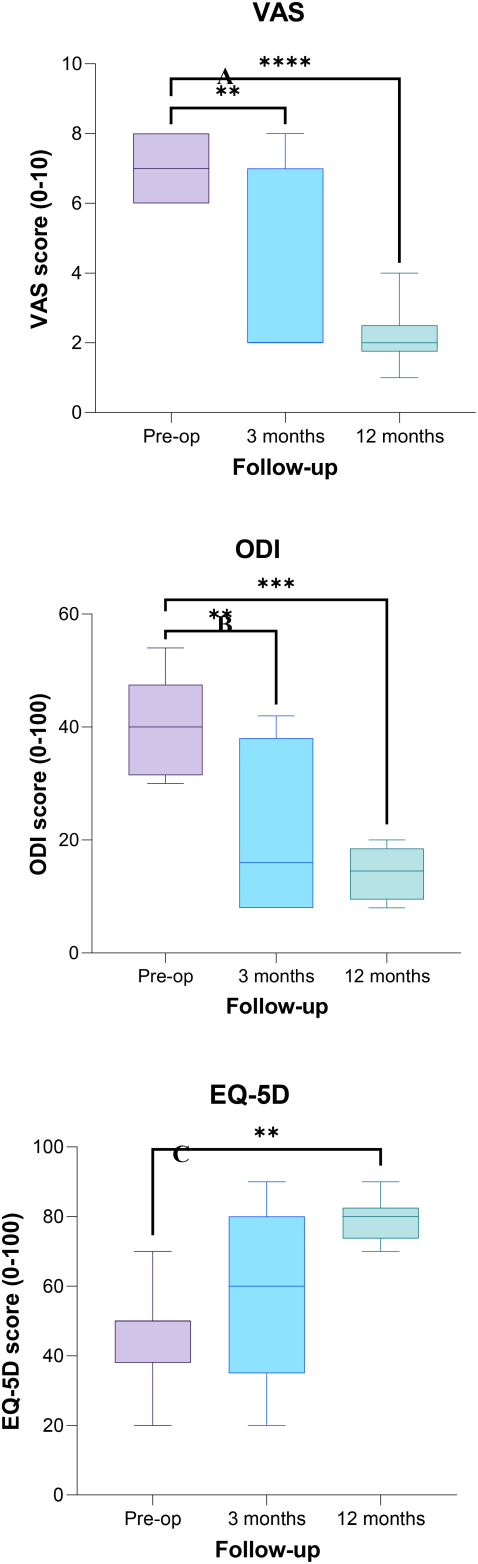
Plot of Visual Analog Scale (VAS) **(A)**, Oswestry Disability Index (ODI) **(B)**, and EuroQoL-5L (EQ-5L) **(C)** scores evaluated preoperatively, at 3- and 12-month follow-ups. The asterisks show a significant difference between post-operative and preoperative values (** *p* < 0.005; *** *p* < 0.0005).

Considering all the patients, the median VAS score at baseline was 7.0, decreasing to 2 at the 3-month follow-up which was further reduced at the 12-month follow-up, with a statistically significant difference between baseline and the 3-month (*p* < 0.005) and 12-month (*p* < 0.0005) follow-up scores (**Figure 4A**). The median ODI value at baseline was 40.0, and it decreased to 16 at the 3-month follow-up and to 14.5 at the 12-month follow-up, with a significant difference between baseline and follow-up scores (*p* < 0.005 and *p* < 0.0005 at the 3- and 6-month follow-up, respectively) (**Figure 4B**). The EQ-5L score at baseline was 50, and it increased to 60 at the 3-month follow-up, and it was 80.0 at the 12-month follow-up, with a significant difference between baseline and the 12-month score (*p* < 0.005) (**Figure 4C**).

## 4 Discussion

Replacement for autograft bone or other bone graft substitutes in instrumented spinal fusion surgery is one of the main clinical needs, especially for complex spinal surgery or revision procedures. Although iliac crest bone autograft is the current gold standard for inducing arthrodesis after spine surgery, it has several drawbacks: 15%–60% of patients complain of donor site pain up to 2 years after surgery (25–27), up to 16.5% of patients experience donor site pain worse than surgery site pain, 30% of patients report donor site numbness, and 15% have difficulty walking due to the donation (27). A retrospective review of the National Surgical Quality Improvement Program database also demonstrated that iliac crest bone grafting increased the rate of postoperative blood transfusions, extended the average operating time, and lengthened the duration of hospital stay (26–28). Given these setbacks, BMA has been developed as an alternative. Several studies have examined the outcomes of iliac crest BMA (in both concentrated and non-concentrated formulations) in combined posterolateral and lumbar interbody fusion (28, 29). As recently reported, spinal fusion surgery BMA can be also easily obtained via a relatively non-invasive method from the vertebral bodies, in the routine course of pedicle screw instrumentation during spine surgery. Published studies using iliac crest BMA or vBMA with different combinations of stabilizers or carriers in spinal surgery are still few and have inconsistent results (28–31). This has previously been attributed to the lack of an effective BMA processing method and a structural texture and to the possibility of dispersion away from the implant site. Recently, for the first time, the potential use of a powerful formulation of BMA, the BMA clot, was described by our team (15, 17, 18). It comprises a clot naturally formed from vertebral bone marrow, which has all the vBMA elements preserved in a matrix molded by the clot. The beneficial effects of the vBMA clot consist in the action exerted by the natural three-dimensional matrix able to deliver osteocompetent cells and biomolecules as well as in the potential anti-inflammatory and antibacterial properties of the clot itself (32–36). To assess safety and efficacy in autologous vBMA clot multifunctional bioscaffold in instrumental posterior lumbar fusion, a limited human pilot study was conducted. Preliminary data from this study demonstrated a satisfactory safety profile in postlateral instrumented fusions evaluated over different follow-ups. No patient displayed systemic toxicity and/or ectopic bone formation. Radiographically, the Brantigan classification showed a fusion success rate of approximately 100% at 12 months of follow-up, considering the levels with C, D, and E grades of the Brantigan classification, as well as a significant increase of bone density by CT from 6 to 12 months of follow-up. To date, no studies have investigated the fusion rate of vBMA or its derivative formulation used alone principally because of the lack of structural integrity. In fact, in all studies in the literature, BMA is always mixed with a carrier such as autograft, allograft, ceramics, or demineralized bone matrix (DBM) prior to implantation. When combining BMA and DBM, a fusion rate of approximately 81% can be achieved (37). The International Spine Study Group described an 87% fusion rate in its retrospective review of anterior lumbar interbody fusions using mineralized collagen and BMA (37). Hart et al. evaluated 80 patients—40 received allograft chips alone (40% success rate) and the other 40 received allograft chips mixed with BMA (80% success rate)—and emphasized the ability of BMA to increase the posterolateral lumbar fusion (38). In this study, a higher fusion rate was obtained when autologous vBMA was used alone, thus proving to be a safe, sustainable, high-quality, and patient-friendly treatment that does not need any additional surgical time, other donor site involvement, *ex-vivo* cell manipulation, stabilizers or carriers, synthetic induction factors, biomaterials and/or scaffold, and complex fabrication processes. The results of this study were also accompanied by excellent clinical results. The ODI, VAS, and EQ-5L clinical parameters, adopted to measure patient’s disability, pain, and quality of life and measured as change from baseline values to 3 months and then at 12 months, showed a significant improvement, already at 3 months of follow-up. This improvement also at 12 months of follow-up is closely correlated to a solid fusion achieved, thus proving the effectiveness of vBMA in achieving spinal fusions.

The safety profile of the vBMA clot is further proven by the lack of adverse events related to its use. In fact, in the present study, except for one event related to postoperative cardiac complications, no other adverse events were registered.

In a recent publication, Heck et al. (39) predicted that the incidence rate of posterior spinal fusion was expected to greatly increase for both women and men: a 246% increase in the total number of posterior spinal fusions for women 75 years and older and a 296% increase for men 75 years and older. The authors emphasize the need to focus on frailty research as well as adequate financial and human resource allocation to make this future surgical procedure viable for the health systems. Assuming this to be the case, the use of vBMA clot can be viewed as a “one-step” procedure, to achieve fusion without any assistance from biomaterials, making it easily accessible, purchasable, and therefore, highly sustainable.

The limitations of the study stemmed from the low number of subjects enrolled, which was exacerbated by the worldwide COVID-19 pandemic that severely reduced surgical activities during 2020–2021. Moreover, the study population was heterogeneous in terms of the type of intervention, with posterior osteotomies that reduce stability and anterior cages that increase stability. However, in the limited follow-up period considered, the stability was maintained by the construct with screws and rods, and fusion was good in all the cases analyzed. Thus, the results from this pilot study permitted the provision of reliable considerations in terms of performance and safety of the vBMA clot in comparison to other graft substitutes used alone or in combination with unclotted BMA. Taking the limitations reported into consideration, a randomized controlled trial has been planned and is ongoing to examine the effect of vBMA clot in spinal fusion procedures on a wider and more homogeneous population.

## Data availability statement

The raw data supporting the conclusions of this article will be made available by the authors, without undue reservation.

## Ethics statement

The studies involving humans were approved by Comitato Etico Area Vasta Emilia Centro (CE-AVEC), Italy. The studies were conducted in accordance with the local legislation and institutional requirements. The participants provided their written informed consent to participate in this study.

## Author contributions

Conception and design of the work: GB, FS, MF. Acquisition and analysis of data for the work: GB, FS, GT, MS, CG, PS, PR, RG. Writing draft of the paper: FS. Revision of paper for publication: GB, CG, GT. Supervision: AG, GG. All authors contributed to the article and approved the submitted version.
